# A217 OUTCOMES OF BALLOON ASSISTED ENDOSCOPIC STRICTURE DILATION IN PATIENTS WITH SMALL BOWEL CROHN’S DISEASE

**DOI:** 10.1093/jcag/gwae059.217

**Published:** 2025-02-10

**Authors:** J C Bowron, M Fazal, M Reeson, B Halloran, S Zepeda-Gomez

**Affiliations:** Medicine, University of Alberta Faculty of Medicine & Dentistry, Edmonton, AB, Canada; Medicine, University of Alberta Faculty of Medicine & Dentistry, Edmonton, AB, Canada; University of Alberta, Edmonton, AB, Canada; Medicine, University of Alberta Faculty of Medicine & Dentistry, Edmonton, AB, Canada; Medicine, University of Alberta Faculty of Medicine & Dentistry, Edmonton, AB, Canada

## Abstract

**Background:**

Crohn’s Disease (CD) is a subset of inflammatory bowel disease (IBD) that has unique pharmacologic, endoscopic and surgical considerations secondary to chronic, transmural inflammation that affects the entire gastrointestinal tract. The natural history of this disease is complicated by formation of fibro-stenotic strictures, adhesions and penetrating fistulae. Balloon-assisted endoscopy (BAE) has significantly improved the ability to assess and treat small bowel CD.

**Aims:**

We present a Canadian cohort of patients that have undergone BAE for stricture dilation of fibrostenotic CD. We retrospectively analyzed demographic, clinical and endoscopic risk factors that could be associated with increased risk of requiring surgical management.

**Methods:**

Retrospective analysis of 157 patients undergoing BAE for stricture dilation of known CD from Apr 2012 – Jan 2024 at the University of Alberta Hospital. A total of 282 procedures were performed and 629 strictures dilated. Patients were then subdivided into two groups: patients that required surgery at any point after their first BAE compared with patients managed with balloon-assisted endoscopic stricture dilation alone.

**Results:**

Of 157 patients with confirmed stricturing CD, 65/157 (41.4%) required surgery between Apr 2012- Jan 2024 and 92/157 (58.6%) patients were managed with endoscopic intervention. The average age at the time of their first BAE was 55.9 years and mean disease duration was 20.76 years in the surgery group. Average patient age in the non-surgical group was 52 years and mean disease duration was 17.9 years. 43.08% (28/65) of patients were active smokers in the surgical group vs. 26.09% (24/92) in the non-surgical group. Concurrent or previous biologic use was comparable at 76.9% and 73.9% in the surgical and non-surgical groups, respectively. The average minimum diameter dilated was 15.2(+/- 0.25) mm in the surgical group vs. 16.2(+/- 0.16) mm in the non-surgical group. The incidence of non-traversable strictures post-dilation was higher in the surgical group (34/231, 14.72%) compared with the non-surgical group (16/398, 9.20%).

In total, 97 surgeries were performed and average time to surgery from first BAE was 32.22 months (excluding emergent/urgent surgeries). Average time from most recent BAE to surgery was 8.79 months. There were 3 emergent surgeries (<24 hours from BAE to surgery, including one perforation) and 7 urgent surgeries (<30 days from BAE to surgery).

**Conclusions:**

BAE has revolutionized endoscopic management of small bowel stricturing CD. Based on our retrospective case cohort, most patients with symptomatic strictures in the small bowel secondary to CD can be successfully managed with balloon-assisted endoscopic dilation. BAE may also play an important role in preventing emergent/unplanned surgical interventions.

**Funding Agencies:**

None

